# Better Informing Decision Making with Multiple Outcomes Cost-Effectiveness Analysis under Uncertainty in Cost-Disutility Space

**DOI:** 10.1371/journal.pone.0115544

**Published:** 2015-03-09

**Authors:** Nikki McCaffrey, Meera Agar, Janeane Harlum, Jonathon Karnon, David Currow, Simon Eckermann

**Affiliations:** 1 Flinders Clinical Effectiveness, Flinders University, Bedford Park, South Australia, Australia 5041; 2 Department of Palliative Care, Braeside Hospital, Prairiewood, New South Wales, Australia; 3 South Western Sydney Local Health District, Liverpool, New South Wales, Australia; 4 School of Population Health and Clinical Practice, University of Adelaide, Adelaide, South Australia, Australia; 5 Palliative and Supportive Services, Flinders University, Bedford Park, South Australia, Australia; 6 Centre for Health Service Development, Australian Health Services Research Institute, University of Wollongong, Wollongong, New South Wales, Australia; National Institute for Public Health and the Environment, NETHERLANDS

## Abstract

**Introduction:**

Comparing multiple, diverse outcomes with cost-effectiveness analysis (CEA) is important, yet challenging in areas like palliative care where domains are unamenable to integration with survival. Generic multi-attribute utility values exclude important domains and non-health outcomes, while partial analyses—where outcomes are considered separately, with their joint relationship under uncertainty ignored—lead to incorrect inference regarding preferred strategies.

**Objective:**

The objective of this paper is to consider whether such decision making can be better informed with alternative presentation and summary measures, extending methods previously shown to have advantages in multiple strategy comparison.

**Methods:**

Multiple outcomes CEA of a home-based palliative care model (PEACH) relative to usual care is undertaken in cost disutility (CDU) space and compared with analysis on the cost-effectiveness plane. Summary measures developed for comparing strategies across potential threshold values for multiple outcomes include: expected net loss (ENL) planes quantifying differences in expected net benefit; the ENL contour identifying preferred strategies minimising ENL and their expected value of perfect information; and cost-effectiveness acceptability planes showing probability of strategies minimising ENL.

**Results:**

Conventional analysis suggests PEACH is cost-effective when the threshold value per additional day at home (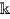
_1_) exceeds $1,068 or dominated by usual care when only the proportion of home deaths is considered. In contrast, neither alternative dominate in CDU space where cost and outcomes are jointly considered, with the optimal strategy depending on threshold values. For example, PEACH minimises ENL when 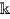
_1_=$2,000 and 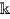
_2_=$2,000 (threshold value for dying at home), with a 51.6% chance of PEACH being cost-effective.

**Conclusion:**

Comparison in CDU space and associated summary measures have distinct advantages to multiple domain comparisons, aiding transparent and robust joint comparison of costs and multiple effects under uncertainty across potential threshold values for effect, better informing net benefit assessment and related reimbursement and research decisions.

## Introduction

Conventional cost-effectiveness analysis (CEA) is limited to consideration of one measure of effect such as life years gained [1]. However, when multiple outcome domains are important such single outcome comparison can lead to conflicting conclusions concerning preferred strategies [2, 3]. Consequently, decisions about the costs and benefits of funding allocations can be misinformed and lead to inefficient distribution of finite health care resources [2, 4].

The widely applied quality-adjusted life year (QALY) measure overcomes this limitation to the extent that impacts on multiple domains of health can be integrated with survival [1]. However, QALYs calculated using generic multi-attribute utility instruments (MAUIs) such as the EQ-5D [5] focus upon health alone as the sole indicator of value. Empirical studies have shown that individuals may also gain utility from factors relating to the provision of health care [6–8]. Economic analyses focusing on health alone do not enable robust coverage of the impacts of interventions in complex disease areas where multiple, diverse domains of effect are important. For example, in palliative care, many patients obtain value from receiving home-based rather than hospital care [9].

In palliative care, the use of the QALY measure is also limited by the:

1. omission of important patient-valued domains from generic MAUIs, such as preparation for death and existential issues [10, 11];
2. changes in patient and clinical perspective due to the proximity of death when valuing utility;
3. dearth of palliative care specific utility instruments incorporating the key domains important to patients receiving palliative care [12];
4. limited availability of suitable palliative care quality of life (QOL) measurement tools to ‘map’ to common MAUIs; and
5. the inability to integrate the impacts on carers’ utility, survival and willingness to care [3, 13, 14].

One option might be to develop a condition-specific MAUI to incorporate palliative care-related effects of interest. Naturally, this would require identifying salient health and non-health related QOL domains and items for inclusion within each domain. Furthermore, confirmation of the content and construct validity and responsiveness of the condition-specific MAUI would be necessary [15]. However, such research would be expected to take years to complete and still faces issues of how to weight or combine effects to inform societal decision making with the objective of maximising budget constrained net benefit (NB) across research, reimbursement and regulation decisions [16]. Alternatively, cost-consequences analysis (CCA) [1] with disaggregated mean costs and multiple outcomes explicitly presented has been advocated as a preferred method in these situations [17, 18]. However, in CCA, cost and multiple outcomes are treated separately without consideration of their interaction or joint uncertainty.

As Briggs et. al. argue in seminal papers on the death of cost-minimisation [19], and cost effectiveness under uncertainty [20], separate and sequential hypothesis tests on differences in outcomes and costs lead to fallacious inferences. It is important that CEA represents joint uncertainty associated with cost and outcomes so that funders and policy makers can make valid inferences and optimise across joint research, reimbursement, regulation and pricing decisions [16, 21, 22]. Jointly evaluating costs with multiple effects and their potential values under uncertainty would enable robust and transparent trade offs between impacts of strategies and the NB of strategies under uncertainty and hence aid consideration of the consequences of reimbursement decisions. Further, if summary measures representing the expected value of perfect information were developed, these measures could start to address decisions related to the value of conducting future research relative to the cost of obtaining the additional information [16, 21, 23]. Ultimately, economic evaluations can easily misrepresent the relative NB of palliative and end-of-life care without simultaneous consideration of costs and multiple outcome domains under uncertainty. Accessible, robust and generalisable methods for jointly comparing cost and multiple outcomes under uncertainty consistent with budget constrained maximisation of NB are needed to better inform funding decisions in such settings.

For multiple strategy comparisons Eckermann et al. [24–26] demonstrate distinct decision making advantages of: (i) presenting costs and effects on the cost-disutility (CDU) plane, with effects framed from a utility reducing perspective, e.g. mortality, morbidity or reduction in QALYs, and flexible axes where costs and effects are measured relative to the least costly and most effective strategies respectively; and (ii) simply calculated expected net loss (ENL) curves and frontiers for each strategy which identify both the optimal strategy in minimising ENL at any threshold value and their expected value of perfect information (EVPI). This paper extends these methods to consider multiple outcomes under uncertainty and whether and how such comparison can be used to better inform societal decision making when optimising budget constrained NB.

The next section summarises conventional CCA and CEA applied to compare multiple outcomes with alternative palliative care models. The new methodology, multiple outcomes comparison in CDU space, and associated summary measures are then introduced and illustrated. The relative merits of these alternative methods for multiple outcomes CEA are discussed in palliative care settings and more generally with multi-criteria decision analysis (MCDA) and finally conclusions and implications are drawn.

## Methods

The cost effectiveness of a home-based palliative care model (Palliative Care Extended Packages at Home (PEACH)) which aimed to expedite discharge and enable patients to remain at home is evaluated relative to usual care using participant-level data from a pilot study. Full details of the economic evaluation are presented elsewhere [27]. Ethics approval for the pilot study was granted by Sydney South West Area Health Service Human Research Ethics Committees. Written informed consent was obtained from the pilot study participants.

### Cost-consequences and cost-effectiveness analyses

Mean incremental per patient effects and net costs were calculated for PEACH relative to usual care including: days at home; place of death; PEACH intervention costs; costs of specialist palliative care service use; and costs of acute hospital and palliative care unit inpatient stays and outpatient visits. Net benefit (Equation 1) was calculated to address analytical and inferential shortcomings of the incremental cost-effectiveness ratio (ICER), while retaining the same underlying cost effectiveness objective [20, 28–31]. Under the NB approach, an intervention is considered cost- effective if, at specified decision-maker threshold values, the monetary value of the incremental effects is greater than the incremental costs i.e., incremental net benefit (INB) is positive (see Equation 2) [24]. The intervention with the greatest INB is considered the most cost-effective in multiple intervention comparison. In budget constrained health systems, threshold values for effects should be the health shadow price for a unit gain of effect reflecting opportunity cost and maximisation of health system outcomes, i.e. represent the best alternative way of achieving the same effects [22, 32–34].

$$NB_{1}=𝕜\times E_{1}-C_{1}$$ Equation 1

$$𝕜(E_{1}-E_{0})-(C_{1}-C_{0})>0$$ Equation 2

where, 1 = evaluated intervention, 0 = comparator, *C* = cost, *E* = effectiveness and *k* = the threshold value.

INB and cost-effectiveness acceptability curves (CEACs) were estimated at potential threshold values for one extra day at home, the primary outcome [31, 35]. Uncertainty for costs, effects and cost effectiveness were estimated bootstrapping on participants’ costs and effects pairs across 10,000 replicates.

### Comparison on the cost-disutility plane

#### Net benefit correspondence theorem (NBCT)

Eckermann [36] and Eckermann, Briggs and Willan [24] demonstrated a one to one correspondence between (i) maximising NB and (ii) minimising costs and the decision maker’s value of events from a utility-reducing perspective. Applying this approach, the equation for INB (Equation 2) [28] is transformed into the following,

$$INB_{*i}=(𝕜\times DU_{i}+C_{i})-(𝕜\times DU_{*}+C_{*})$$ Equation 3

where *DU* are effects framed from a disutility perspective, *C* represents costs, *i* represents the strategy under consideration and * is the optimal strategy at threshold value for a unit of effect.

#### Radial efficiency measures on the CDU plane

Reframing effects from a utility-reducing perspective and comparing strategies on the CDU plane allows NB improvement with contraction to the vertex; that is performance improves with equi-proportionally contraction of costs and effects to the origin. Importantly, these radial properties enable efficiency measures invariant to scale of axes, unlike non-radial efficiency measures [37] and allow conventional economic, technical and allocative efficiency measures [22–24, 29] to be calculated using standard frontier estimation methods such as data envelopment analysis (DEA) or index methods. Generally, employing input-orientated DEA, a piecewise, convex, inner-boundary is formed reflecting combinations of multiple inputs that cannot be proportionally contracted with the feasible set (convex combination of other strategies) [38].

**Fig 1:**
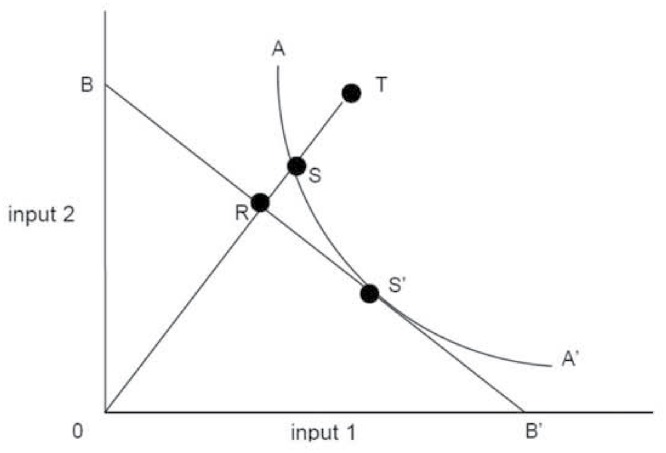
Farrell’s production possibilities frontier for two inputs and one output (adapted from Coelli [41].

Farrell [37], in 1957, also showed radial properties enable economic efficiency to be expressed as the product of technical and allocative efficiency [37]. Hence when factor prices are applied [38] efficiency can be calculated [24, 26, 36] and decomposed into technical and allocative efficiency components. In Fig. 1, BB’ represents possible combinations of inputs for a given cost. S’ represents an allocatively efficient point because this lies on the production possibilities frontier with minimal cost given factor prices, i.e. is using the appropriate mix of inputs given input prices to produce the given output. Allocative *inefficiency* at point T is represented by RS/0S. That is, the amount by which production costs can be reduced if the ratio of inputs used by the firm at point T were identical to those used by the firm at point S’, the technically and allocatively efficient firm [37].

Traditionally results presented graphically on the cost-effectiveness plane use a fixed comparator with improved performance indicated by south-east movement. Further, with multiple strategy comparison, the point of reference changes along the efficiency frontier [24]. The use of standard frontier estimation methods are prevented on the cost-effectiveness plane by the absence of radial properties.

For multiple strategies with a *single* effect, Eckermann et al [24, 25] show that comparison at potential threshold value for effects on the CDU plane naturally leads to considering the net loss (NL) statistic for strategies, the expected net loss (ENL) relative to the strategy maximising NB, ENL curves for each strategy and the ENL frontier as the lower bound of ENL curves.

In this paper use of DEA in CDU space is also shown to facilitate explicit and robust consideration of the interaction of uncertainty between costs and *multiple* outcomes and lead to useful summary measures including:

1. Threshold regions, combinations of threshold values for joint outcomes where alternative service models maximise NB [38].
2. ENL planes which quantify differences in ENL [28] across threshold values for multiple outcomes, (i) time at home *and* (ii) death at home rather than in an inpatient setting;
3. The ENL contour which identifies the service model that minimises ENL across bootstrapped replicates for combinations of threshold values across multiple outcomes [24, 25]; and
4. Cost-effectiveness acceptability planes which show the probability of each service model minimising ENL at given combinations of value of effects for multiple outcomes.

### Comparison in cost-disutility space

#### Technical efficiency frontier

The technical efficiency frontier was constructed using DEA with a Farrell input-orientated technical efficiency model run under constant returns to scale [39]. Mean incremental cost relative to the cheapest model of care and mean incremental effects relative to the most effective model of care framed from a disutility perspective were included as strongly disposable inputs. The resulting frontier identifies service models that minimise cost for given effects framed from a disutility perspective [24–25, 36]. A technical efficiency score of one indicates that service models form part of the technical efficiency frontier and minimise costs for given effect rates at some combination of threshold values. Technical efficiency scores less than one indicate the model of care is dominated or extended dominated by the other strategies and does not form part of the frontier, i.e. is technically inefficient. In other words, both cost and disutility of effects can be equi-proportionally reduced relative to a given strategy or convex combinations of other strategies.

#### Deterministic cost effectiveness analyses

To determine threshold regions where alternative models of care are preferred, NL is first calculated from incremental analysis in CDU space. Applying the net benefit correspondence theorem [24], the objective of maximising NB is equivalent to minimising NL. The NL of any given model of care (*i*) is the loss in NB from choosing *i* rather than the optimal model of care (*) at given threshold value *k* for one effect and can be found as follows:

$$NL_{*i}=(𝕜\times DU_{i}+C_{i})-(𝕜\times DU_{*}+C_{*})$$ Equation 4

where *DU* is the outcome framed from a utility-reducing perspective and *C* represents costs.

This relationship can be extended to include multiple outcomes [38]. For example, for two outcomes and two strategies, *i* and *j*:

$$NL_{*i}=(({𝕜}_{1}\times DU_{i1})+({𝕜}_{2}\times DU_{i2})+C_{i})-(({𝕜}_{1}\times DU_{*1})+({𝕜}_{2}\times DU_{*2})+C_{*}$$ Equation 5

$$NL_{*j}=(({𝕜}_{1}\times DU_{j1})+({𝕜}_{2}\times DU_{j2})+C_{i})-(({𝕜}_{1}\times DU_{*1})+({𝕜}_{2}\times DU_{*2})+C_{*}$$ Equation 6

With deterministic analysis the preferred model of care is the model which minimises mean NL at any given combination of threshold values, i.e. *C + DU*_1_𝕜_1_ + *DU*_2_𝕜_2_ is minimised. Therefore, strategy *i* is preferred to strategy *j* when the mean NL of *i* is lower than the mean NL of *j*, and vice versa. To find the regions where alternative models of care are preferred, the boundary of the regions is first determined by equating the NL expressions for adjacent compared strategies on the frontier in CDU space, and solving for 𝕜_1_ and 𝕜_2_, i.e.

$$C_{i}+({𝕜}_{1}\times DU_{i1})+({𝕜}_{2}\times DU_{i2})=C_{j}+({𝕜}_{1}\times DU_{j1})+({𝕜}_{2}\times DU_{j2})$$ Equation 7

Values either side of the boundary readily identify the combinations of potential threshold values where each model minimises the mean NL.

#### Stochastic cost-effectiveness analyses (accounting for joint uncertainty)

Bootstrapping methods allow modelling of uncertainty across the joint distribution of incremental costs and multiple incremental outcomes from the participant-level data, allowing for covariance between costs and effects [40]. The following measures are calculated using the bootstrap replicates and summarise the expected return on investment and risk of return across potential threshold values for multiple outcomes given current uncertainty.

Choosing a strategy that does not minimise the NL incurs an incremental NL relative to the optimal strategy. For each service model, at a given set of threshold values the NL relative to the NB maximising strategy in each replicate is calculated and averaged across 10,000 replicates to estimate ENL [24]. An expected loss arises for the proportion of replicates in which the service model does not maximise the NB at the specified threshold values, reflecting decision uncertainty given current trial evidence.

As in the case of ENL curves for a single effect [22], ENL planes quantify differences in ENL across models of care for different combinations of values for effects. ENL planes are formed by varying the threshold values for the multiple effects and re-calculating the average ENL across replicates for each model of care. The distance between planes represents the difference in ENL between models of care at any set of threshold values for effects under uncertainty [24].

The contour is formed by the lower bound of the ENL planes across models of care, i.e. the lowest ENL values at combinations of threshold values are determined from consideration of both planes, analogous to the ENL frontier as the lower bound of ENL curves [16, 24]. The contour readily identifies the service model that minimises ENL for any set of values [22] for 𝕜_1_ and 𝕜_2_in the case of the PEACH study.

The expected value of perfect information (EVPI) is the loss from a bad decision that could be avoided with perfect, rather than current information [21]. As with the ENL frontier, the ENL contour naturally represents the EVPI per patient associated with choosing the strategy minimising ENL [16, 24] given current uncertainty, but as a function of threshold values for multiple rather than single effects.

Cost-effectiveness acceptability planes (CEAPs) show the probability that each model of care minimises ENL conditional on threshold values for multiple effects. For each model of care, the CEAP is formed by determining the proportion of replicates that minimise ENL for different combinations of threshold values for effects.

## Results

### Cost-consequences and cost-effectiveness analyses

A summary of the trial-based incremental costs and consequences framed from a utility perspective are presented in Table 1. INB curves and CEACs are presented in McCaffrey et al [27]. PEACH is the preferred service model when the threshold value for one extra day at home (𝕜_1_) exceeds $1,068 as the value of expected incremental benefits exceeds expected incremental costs. However, the CCA suggests PEACH is dominated by usual care when the proportion of home deaths is considered.

**Table 1:** Summary of incremental costs and outcomes framed from a utility perspective at 28 days for PEACH versus usual care.

	PEACH	Usual Care	Increment
	(n = 23)^1^	(n = 8)	
**Consequences, mean (95% CI^2^)**			
Number of days at home	13.09	12.13	0.96
	(8.52, 17.65)	(5.88, 18.38)	(-6.79, 8.64)
Proportion of participants who died, %	69.57	62.50	7.07
	(52.17, 86.96)	(25.00, 100)	(-45.11, 30.43)
Of those who died, the proportion of home deaths %	56.25	80.00	-23.75
	(31.25, 80.00)	(33.33, 100)	(-63.16, 25.00)
**Costs, mean (95% CI^2^)**			
PEACH	$3,489	0	$3,489
	($2,170, $4,943)		($2,170, $4,943)
Specialist palliative care services	$361 ($256, $470)	$372 ($229, $526)	-$11 (-$196, $168)
Inpatient stay^3^	$2,603	$5,053	-$2,450
	($1,205, $4,147)	($2,084, $8,139)	(-$5,843, $957)
**Total**	$6,452	$5,425	$1,027
	($,4,469, $8,586)	($2,404, $8,531)	(-$2,612, $4,738)
**Threshold value** ^**4**^ **above which the mean INB becomes positive (95% CI)**			**$1,068**
			(-$6,627, $6,578)

^1^ one participant in the PEACH arm was excluded from the analysis due to incomplete cost data;

^2^ calculated with bootstrap analysis;

^3^ hospital or palliative care unit;

^4^ for one extra day at home

### Analysis in cost-disutility space

A summary of the trial-based incremental costs and consequences framed from a utility-reducing perspective are presented in Table 2. The mean incremental cost relative to the cheapest model of care and mean incremental effects framed from a disutility perspective relative to the most effective model of care are calculated for each service model. For example, when considering incremental inpatient days, the mean incremental effect for PEACH is zero because PEACH is the most effective model of care (14.9–14.9). Similarly, as usual care is the cheapest model of care, the mean incremental cost for usual care is zero ($5,425—$5,425).

**Table 2:** Summary of incremental costs and outcomes framed from a disutility perspective at 28 days for PEACH versus usual care.

	Model of care		Increment	
	PEACH	Usual Care	PEACH	Usual Care
	(n = 23)^1^	(n = 8)		
**Consequences, mean (95% CI^2^)**				
Number of inpatient days	14.91	15.88	0^3^	0.96^3^
	(10.35, 19.48)	(9.63, 22.13)	(0, 6.78)	(0, 8.64)
Of those who died, the proportion of inpatient deaths, %	43.75	20.00	23.75^3^	0^3^
	(20.00, 68.75)	(0, 66.67)	(0, 63.16)	(0, 25.00)
**Costs, mean (95% CI^2^)**	$6,452	$5,425	$1,027^4^	$0^4^
	($4,469, $8,586)	($2,404, $8,531)	(0, $4,738)	(0, $2,612)

^1^ one participant in the PEACH arm was excluded from the analysis due to incomplete cost data;

^2^ calculated with bootstrap analysis;

^3^ relative to the most effective model of care (*DU*_1*i*_ *-DU*_1*_);

^4^ relative to the cheapest model of care (*C*_*i*_-C_*_)

#### Technical efficiency frontier

**Fig 2:**
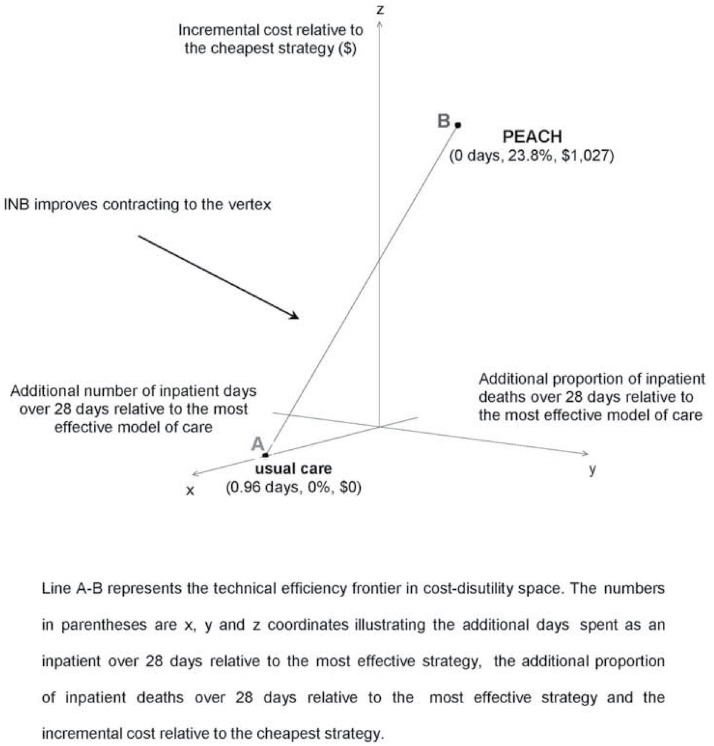
Technical efficiency frontier in cost-disutility space for PEACH and usual care.

Both models of care are part of the technical efficiency frontier in CDU space where costs and multiple outcomes are considered (Fig. 2), i.e. both models of care minimise NL for some set of threshold values for time spent at home and place of death. The frontier in CDU space (line A-B in Fig. 2) represents the inner bound of linear combinations of service models closest to the origin, i.e. those minimising ENL at different combinations of threshold values for effects.

#### Threshold regions

Following the incremental analysis in CDU space (Table 2), the mean NL for each model of care can be expressed as follows (see Equation 7):

- Usual care, *NL* = $0 + 0.96𝕜_1_ + 0𝕜_2_ = 0.96𝕜_1_; and
- PEACH, *NL* = $1,027 + 0𝕜_1_ + 0.24𝕜_2_ = $1,027 + 0.24𝕜_2_

Where 𝕜_1_ is the threshold value for one extra day at home over 28 days and 𝕜_2_ is the threshold value for one extra home death over 28 days.

**Fig 3:**
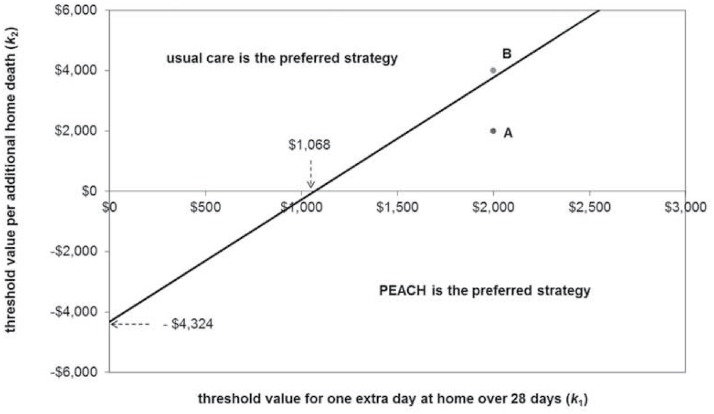
Threshold regions over which each service model is preferred.

Equating the NL expressions determines the boundary between regions of threshold value combinations where each model of care is preferred, represented by the line in Fig. 3.

PEACH is preferred to usual care when the mean NL of PEACH is lower than the mean NL of usual care, i.e. $1,027 + 0.24𝕜_2_ < 0.96𝕜_1_, simply rearranged to 𝕜_1_ > 0.25𝕜_2_ + $1,068. Hence, for example when threshold values for both an extra day at home (𝕜_1_) and home deaths (𝕜_2_) are $2,000, PEACH minimises NL and is the preferred model of care; point A in Fig. 3. Conversely, when 𝕜_1_ = $2,000 and 𝕜_2_ = $4,000, usual care is preferred (point B, Fig. 3). This highlights that neither alternative dominated the other in CDU space when incremental cost, days at home *and* the proportion of home deaths are jointly considered.

#### Expected net loss planes

**Fig 4:**
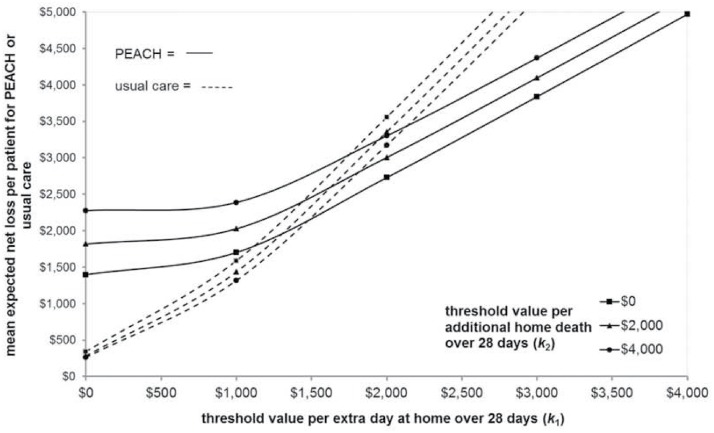
Expected net loss planes (two dimensional representation).

Fig. 4 shows a two dimensional representation of the ENL planes for PEACH and usual care. The threshold value per extra day at home is represented along the x-axis and the mean ENL per patient on the y-axis. Using the previous example, when 𝕜_1_ = $2,000 and 𝕜_2_ = $2,000, PEACH minimises the ENL with an average loss in expected NB of $3,004 per participant. This loss arises as there are a proportion of replicates (4,838/10,000) in which PEACH does not maximise the NB at these threshold values. At these same values the ENL per patient if usual care is adopted is $3,354. If 𝕜_1_ = $2,000 and 𝕜_2_ = $4,000 the ENL per patient increases to $3,301 for PEACH and reduces to $3,172 for usual care. The distance between planes represents the difference in per patient ENL between models of care at any set of threshold values for effects under uncertainty [24]. For example, when 𝕜_1_ = $2,000 and 𝕜_2_ = $2,000 the mean ENL per patient if PEACH is adopted is $3,004 and $3,354 if usual care is chosen, with a difference in ENL of $350 per patient.

#### Expected net loss contour

**Fig 5:**
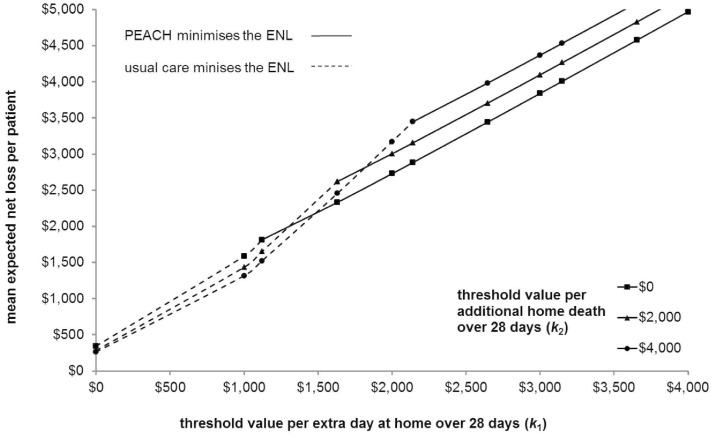
Expected net loss contour (two dimensional representation).

Fig. 5 is a two dimensional representation of the ENL contour which is formed by the lower bound of the ENL planes across models of care (see Figs. 4 and 5) [16, 24]. The dotted line indicates the combinations of 𝕜_1_ and 𝕜_2_, where usual care minimises the ENL, whereas the solid line illustrates combinations of 𝕜_1_ and 𝕜_2_ where PEACH minimises the ENL. Using the previous example, if 𝕜_1_ = $2,000 and 𝕜_2_ = $2,000, PEACH minimises the ENL at $3,004 per participant across 10,000 replicates. When 𝕜_1_ = $2,000 and 𝕜_2_ = $4,000, usual care minimises the ENL. Furthermore, in the latter scenario choosing usual care minimizes ENL at $3,172 but given current uncertainty, PEACH minimizes ENL in 4,905/10,000 replicates. This loss of $3,172 per participant from choosing usual care would be avoided with perfect information as the decision-maker would be able to pick the service model minimizing ENL in each realisation.

#### Cost-effectiveness acceptability planes

**Fig 6:**
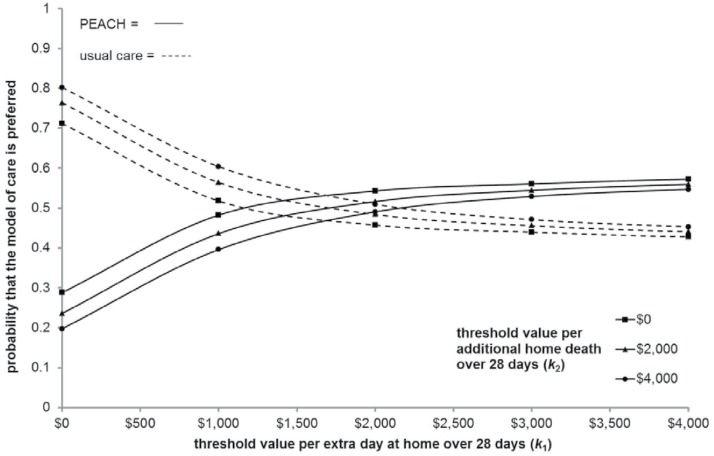
Cost-effectiveness acceptability planes (two dimensional representation).

Fig. 6 shows the CEAPs which represents the probability that the strategy under consideration minimizes ENL at given combinations of value of effects for multiple outcomes. For example, when 𝕜_1_ = $2,000 and 𝕜_2_ = $4,000 there is a 49.05% chance that PEACH is the preferred option because PEACH minimizes ENL in 4,905/10,000 replicates.

## Discussion

When assessing the cost effectiveness of interventions with multiple outcomes, presentation in CDU space and use of ENL planes and contours to summarise cost effectiveness has been illustrated to overcome limitations of conventional CEA and CCA analysis. Partial analysis, where individual outcomes and costs are considered separately, suggested PEACH is associated with higher costs and increased benefits over 28 days relative to usual care when time spent at home was the sole measure considered. However, PEACH was dominated by usual care when the proportion of home deaths alone was considered. These results illustrate that choice of effect where analysis is restricted to single effect substantially alters cost-effectiveness inferences. In comparison, neither service model dominated in CDU space, when incremental cost and multiple outcomes were jointly considered. Compared with CCA the proposed approach has distinct advantages in allowing for joint uncertainty of incremental cost and outcomes, and cost effectiveness. If societal decision making asymptotes to risk neutrality [26, 41] then findings suggest PEACH maximises expected NB when 𝕜_1_ > 0.25𝕜_2_ + $1,068. If preferences are somewhat risk-averse, trade-offs may also arise between minimising ENL and maximising the probability of minimising ENL across compared strategies for discrete regions of threshold values.

This paper extends previous work showing advantages of the CDU plane for comparing multiple strategies. Cost and multiple effects of alternative models of care are clearly presented in CDU space, facilitating identification, presentation and exploration of trade-offs between effects. Collectively, the new summary measures compared expected NB between strategies across any set of feasible threshold values for multiple effects and the impact of joint uncertainty. Threshold regions display the combinations of values over which alternative models of care minimise mean NL or equivalently maximise NB. ENL planes present differences in ENL between alternatives, while the ENL contour simultaneously establishes: (i) the intervention that minimises ENL; and (ii) EVPI with current evidence. Finally, CEAPs estimate the probability that interventions minimise ENL. This may be of particular interest across discrete threshold regions where the model of care with the lowest ENL does not have the greatest probability of minimising ENL if societal decision making remains somewhat risk averse [41]. However, it should be stressed that CEAPs, like CEACs, do not directly identify the strategy that minimises ENL [25], unlike ENL planes and contours. The summary measures presented in this paper allow decision-makers to explicitly trade off expected return on investment with the risk of return at different relative and absolute threshold values for multiple outcomes.

Table 3 contrasts and summarises suggested graphical representation and summary measures for informing societal decision making when comparing two strategies and multiple strategies with single and multiple effects.

**Table 3:** Best presentation and summary measures for informing risk-neutral and somewhat risk averse decision making [25].

	Risk preferences	
Scenario	Risk-neutral^#^	Somewhat risk averse^^^
Two strategies & one effect	CE plane	CE plane
	INB curve	INB curves & CI
	ENL curves & frontier	CEA curve
		ENL curves & frontier
Two strategies & multiple effects	Frontier in CDU space^$^	Frontier in CDU space^$^
	Threshold regions	Threshold regions
	ENL planes and contour	ENL planes and contour
		CEAP
Multiple strategies & a single effect	CDU plane	CDU plane
	ENL curves & frontier	ENL curves and frontier
		Trade-offs in discrete threshold regions where they arise between minimising ENL and P(min NL) taken from relevant bilateral CEA curves
Multiple strategies & multiple effects	Frontier in CDU space^$^	Frontier in CDU space^$^
	Threshold regions	Threshold regions
	ENL planes & contour	ENL planes and contour
		Trade-offs in discrete threshold regions where they arise between minimising ENL and P(min NL) taken from relevant bilateral CEAPs

^#^ objective is to maximise ENB;

^^^ trade-offs can arise over discrete threshold regions between the strategy maximising ENB and strategies with a higher probability of maximising NB relative to that strategy;

^$^ for a maximum of two effects; CDU = cost-disutility; CE = cost-effectiveness; CEA = cost-effectiveness acceptability; CEAP = cost-effectiveness acceptability plane; CI = confidence intervals; ENL = expected net loss; INB = incremental net monetary benefit; P(min NL) = probability of minimising net loss

Contemporary economic evaluation methods are limited when comparing interventions with multiple outcomes. Although CCA explicitly presents multiple outcomes and costs, they are considered independently rather than jointly when allowing for uncertainty. Also, this approach does not allow for differences in ENB. Multiple outcomes comparison in CDU space retains advantages of CCA in comparing multiple outcomes while additionally allowing rigorous assessment of joint uncertainty across multiple outcomes and estimation of differences in ENL. Furthermore, the ability of the methods developed to allow for different relative preferences [42] is also valuable for informing individual patient and population level decisions under uncertainty.

Cost-benefit analysis could include multiple effects. However, this approach is limited by the difficulties of valuing health outcomes in monetary terms and the influence of ability to pay on willingness to pay [43, 44]. Multiple outcomes comparison in CDU space allows explicit exploration of relative and absolute monetary values for multiple outcomes on preferred strategies. Further, this approach permits robust analysis of the impact of uncertainty around monetary threshold values with ENL contours.

Cost-effectiveness analysis is limited by a uni-dimensional measure, where choice of outcome can give rise to outcome selection bias as illustrated in this paper. Cost-utility analysis (CUA) is limited by narrowly defined health-related QOL domains in the commonly applied generic MAUIs which exclude important domains unable to be integrated with patient survival in complex disease areas such as palliative care, e.g. utility from preparing for death [45], carer and family impacts and non-health outcomes [46]. The novel methodology presented in this paper enables simultaneous evaluation of such diverse domains of effect.

Previously there have been only a few attempts to develop specific methods to evaluate and present costs and multiple outcomes under uncertainty in an explicit manner. Bjorner and Keiding [47] proposed a relative cost-effectiveness measure. Negrin and Vazquez-Polo [48] presented an alternative Bayesian cost-effectiveness framework. The Bjorner and Keiding approach compares the performance of each intervention relative to the worst performing intervention in a set of interventions using DEA [47]. However, their relative cost-effectiveness measure does not inform decisions between non-dominated interventions, nor allow consideration of stochastic uncertainty, or allow estimation of the probability of maximising expected NB. The Bayesian methodology [48] graphically represents the intervention most likely to maximise NB at various combinations of threshold values for multiple outcomes using a cost-effectiveness acceptability frontier. Geometrically, this is similar to the CEAP derived from comparison in CDU space. However, neither of these previous approaches provides summary measures for quantifying differences in ENB between strategies under uncertainty, which is the critical comparison required to inform risk-neutral or somewhat risk-averse decision making [25, 41].

In this paper, the NBCT is applied with CEA to inform health technology assessment. Similarly, the novel approach to multiple outcome comparison illustrated here could be applied to compare multiple outcomes, objectives and facets of chronic disease management programs [49], health promotion and disease prevention in complex community care settings [50–56]. Multiple aspects relevant to such comparisons might include self-management capability, co-ordination and process aspects of care, network impacts and community uptake and ownership of promotion interventions in complex settings. Other forms of multiple criteria decision analysis (MCDA) have been proposed to allow for multiple objectives and outcomes in some of these settings [57, 58]. The NBCT [24–26] applied to multiple outcomes [59] would enable a comparison of such multiple criteria consistent with maximising NB and is summarised with ENL planes and contours. Unlike CEACs [58] and other transformations proposed, the ENL planes and contour and ENL curves and frontiers [24] are consistent with differences in ENB. This is particularly important given the primary importance under the Arrow-Lind theorem [41] to compare differences in ENB and avoid confounding of relevant probabilities between potentially optimal strategies that arises with multiple strategy CEACs in multiple strategy comparisons [25]. Consequently, application of the proposed methodology in alternative health promotion and prevention settings as well as disease areas, with their associated multiple effects or criteria of interest for decision making are suggested as valuable. Applying the values and preferences of individuals, different patient populations and societal decision makers across jurisdictions for relevant multiple outcomes should be considered in future research to further demonstrate the flexibility of this approach.

If the threshold values for effects in NB are to aid optimization of health outcomes from a fixed budget then assuming the new technology or strategy has net costs the threshold value should reflect the opportunity cost of reimbursement in adopting and financing the new service, technology or strategy [22, 32–34]. That is, the opportunity cost of reimbursing a new technology is the most cost-effective expansion of existing services financed by contraction or displacement of the least cost-effective service. The opportunity cost and threshold value should be estimated allowing for characteristic allocative and displacement inefficiency in health systems where the least cost-effective program in contraction (ICER = *m*) has a higher ICER than the most cost-effective program in expansion (ICER = *n*) and displaced services (ICER = *d*). The health shadow price derived by Pekarsky [34] (see Equation 8), allows for allocative inefficiency (*n < m*) and displacement inefficiency (*d < m*) to reflect the opportunity cost of best alternative adoption and financing actions in reimbursing new technology.

$$\beta_{c}={\left(\frac{1}{n}+\frac{1}{d}+\frac{1}{m}\right)}_{}^{-1}$$ Equation 8

The health shadow price threshold has a value of *n*, equivalent to the ICER of the most cost-effective expansion of current programs if displacement is efficient (*d* = *m*), but is less than *n* where displacement is suboptimal (*d* < *m*), reflecting the potential to improve displacement as well as adoption actions [22]. In the case of multiple effects the associated threshold values should be determined by their relevant health shadow prices as these represent the best alternative way for societal decision makers to achieve each outcome.

Multiple outcomes analysis in CDU space with use of the ENL contour as a summary measure while highlighting which strategy optimises ENB across potential threshold values for effects also naturally represents the expected value of perfect information [24, 25]. This provides a starting point to consider use of value of information (VOI) methods to inform decisions under uncertainty of whether undertaking further research is optimal. VOI methods aid optimisation of trial design in maximising expected return on investment from further research, comparing the expected value relative to expected cost of additional research allowing for relevant decision contexts [16, 21, 60, 61].

### Limitations

Despite distinct advantages over existing methods for multiple outcome comparisons, there are some issues to consider. While multiple outcomes comparison presented in CDU space enables flexible and robust comparison under uncertainty, as with other multiple outcome approaches issues of valuation and trade-offs between outcomes arise. Relative decision-maker threshold values are required for funding or policy decisions in any jurisdiction of interest for their population, practice, and preferences conditional on the budget constrained decision context whether based on cost-effectiveness, cost-utility or cost-benefit analyses. Multiple outcomes comparison in CDU space, unlike alternative methods, enables explicit joint comparison of costs and multiple outcomes prior to valuation and encourages explicit valuation and acknowledgment of trade-offs between outcomes in the decision-making process. These issues are particularly important where the maximand for cost-effectiveness analysis is not stated. In cost-utility analyses, multiple threshold valuation also occurs for different outcomes or domains within the MAUI but is less explicit or readily identified. Generally, valuing effects should, as with the proposed approach, be explicit to enable appropriate translation of impacts to decision making in different contexts.

The methodology presented can be applied to any number of strategies and any number and types of outcome measures. However, whilst graphical presentation for comparison is feasible in two- and three-dimensional space, further dimensions cannot be easily graphically represented in ways that are currently familiar in societal decision-making. Similarly, knowledge of frontier methods such as DEA may be restricted to those with mathematical or economic backgrounds and hence application of these methods to fully exploit the benefits of multiple outcomes comparison in CDU space could require educational support to aid knowledge translation. Despite this, the general formulae promote transferability of the method across different populations and jurisdictions given different values can be imputed for each outcome depending on perspective and context.

Further research is required to exploit the societal decision making advantages of robustly comparing multiple outcomes and multiple strategies in CDU space illustrated in this paper. Application of the approach in alternative disease areas, with different numbers of multiple effects and/or strategies would be particularly valuable. The views of bodies and individuals involved in societal-decision making on the relative merits of alternative presentation and summary measures for evaluating the cost-effectiveness of interventions with multiple effects could also be sought to explore the interface between theoretical and practical advantages and lead to further development of the methodology in practice.

## Conclusion

Better methods are needed to compare the cost effectiveness of alternative strategies with multiple outcomes under uncertainty in complex service delivery areas such as palliative care. On the cost-effectiveness plane analysis is restricted to one outcome and not all outcome domains can be integrated with survival in estimating QALYs. Furthermore, cost-consequences analyses fail to allow for joint uncertainty across outcomes. Comparison in CDU space with ENL planes, contours and CEAPS have been shown to allow presentation of uncertainty across multiple outcomes with summary measures quantifying differences in ENB and the probability of maximising ENB at any given set of threshold values for multiple outcomes. Incorrect inferences are avoided using these presentation and summary measures and risk-neutral or somewhat risk-averse societal decision making better informed, while the potential value of future research (EVPI) is also estimated. In summary, analysis in CDU space provides a readily accessible and systematic way to compare multiple outcomes in CEA and NB assessment under uncertainty. In comparing multiple strategies a more robust picture of the likely trade-offs between costs and benefits, the consequences of funding decisions and the need for future research reduces inferential errors and better informs societal decisions [16, 21, 62, 63].

## Supporting Information

S1 File Raw data. (DOCX)
