# Supplementary material for: Better Informing Decision Making with Multiple Outcomes Cost-Effectiveness Analysis under Uncertainty in Cost-Disutility Space
Source: PLoS One. 2015 Mar 9;10(3):e0115544. doi: 10.1371/journal.pone.0115544 (PMC4353730; doi:10.1371/journal.pone.0115544)
Supplement: S1 File — (DOCX) [file pone.0115544.s001.docx]

**Table S1 Participant-level outcomes for the PEACH pilot study**

| **ID** | **Treatment arm** | **Total days spent in the community*** | **Died during the study period** | **Home death** |
| --- | --- | --- | --- | --- |
| 001 | *PEACH* | 28 | No | No |
| 004 | *PEACH* | 3 | Yes | Yes |
| 005 | *PEACH* | 28 | No | No |
| 007 | *PEACH* | 16 | Yes | No |
| 008 | *PEACH* | 18 | No | No |
| 009 | *PEACH* | 2 | Yes | Yes |
| 010 | *PEACH* | 2 | Yes | No |
| 011 | *PEACH* | 28 | No | No |
| 012 | *PEACH* | 0 | Yes | No |
| 013 | *PEACH* | 28 | No | No |
| 014 | *PEACH* | 4 | Yes | Yes |
| 015 | *PEACH* | 5 | Yes | Yes |
| 017 | *PEACH* | 0 | Yes | No |
| 018 | *PEACH* | 2 | Yes | Yes |
| 019 | *PEACH* | 0 | Yes | No |
| 021 | *PEACH* | 21 | No | No |
| 022 | *PEACH* | 22 | Yes | Yes |
| 023 | *PEACH* | 10 | Yes | Yes |
| 025 | *PEACH* | 2 | Yes | No |
| 026 | *PEACH* | 26 | Yes | Yes |
| 028 | *PEACH* | 28 | No | No |
| 029 | *PEACH* | 23 | Yes | No |
| 031 | *PEACH* | 5 | Yes | Yes |
| 002 | *Usual* | 21 | No | No |
| 003 | *Usual* | 22 | No | No |
| 006 | *Usual* | 2 | Yes | Yes |
| 016 | *Usual* | 25 | No | No |
| 020 | *Usual* | 14 | Yes | Yes |
| 024 | *Usual* | 5 | Yes | Yes |
| 027 | *Usual* | 1 | Yes | No |
| 032 | *Usual* | 7 | Yes | Yes |

*over the 28 day study period

**Table S2 Summary costs for the PEACH pilot study**

| **PEACH** | **Inpatient stay** | **Outpatient** | **Emergency department** | **Specialist services** | **PEACH care package** | **PEACH program** | **Total** |
| --- | --- | --- | --- | --- | --- | --- | --- |
| Grand Totals AU$ | $59,864 | $0 | $0 | $8,295 | $64,904 | $15,339 | ***$148,403*** |
| Average AU$ | $2,603 | $0 | $0 | $361 | $2,822 | $667 | ***$6,452*** |
| **Usual Care** |  |  |  |  |  |  |  |
| Grand Totals AU$ | $40,424 | $0 | $0 | $2,979 | $0 | $0 | ***$43,403*** |
| Average AU$ | $5,053 | $0 | $0 | $372 | $0 | $0 | ***$5,425*** |

**Table S3 Participant-level costs for the PEACH pilot study**

| **ID** | **Treatment arm** | **Specialist Palliative Care** | **Community Nursing** | **Allied Health** | **Equipment** | **1300** | **Total specialist costs** | **Total inpatient costs** | **Total admin costs** | **Total PEACH package** | ***OVERALL COST*** |
| --- | --- | --- | --- | --- | --- | --- | --- | --- | --- | --- | --- |
| 001 | *PEACH* | $159.32 | $45.52 | $50.08 | $0.00 | $0.00 | **$254.91** | **$0** | **$666.92** | **$1,511.10** | ***$2,433*** |
| 004 | *PEACH* | $75.15 | $45.52 | $0.00 | $0.00 | $0.00 | **$120.67** | **$0** | **$666.92** | **$103.35** | ***$891*** |
| 005 | *PEACH* | $447.42 | $273.12 | $0.00 | $0.00 | $0.00 | **$720.54** | **$0** | **$666.92** | **$5,778.45** | ***$7,166*** |
| 007 | *PEACH* | $300.60 | $250.36 | $24.23 | $132.00 | $11.38 | **$718.57** | **$6,066** | **$666.92** | **$8,813.91** | ***$16,265*** |
| 008 | *PEACH* | $120.67 | $273.12 | $0.00 | $0.00 | $0.00 | **$393.79** | **$7,110** | **$666.92** | **$1,665.00** | ***$9,836*** |
| 009 | *PEACH* | $59.26 | $45.52 | $87.63 | $0.00 | $0.00 | **$192.41** | **$0** | **$666.92** | **$134.20** | ***$994*** |
| 010 | *PEACH* | $45.52 | $68.28 | $0.00 | $0.00 | $0.00 | **$113.80** | **$1,808** | **$666.92** | **$490.00** | ***$3,079*** |
| 011 | *PEACH* | $150.30 | $250.36 | $150.23 | $0.00 | $0.00 | **$550.89** | **$0** | **$666.92** | **$0.00** | ***$1,218*** |
| 012 | *PEACH* | $0.00 | $0.00 | $0.00 | $0.00 | $0.00 | **$0.00** | **$9,440** | **$666.92** | **$0.00** | ***$10,107*** |
| 013 | *PEACH* | $104.78 | $569.01 | $0.00 | $0.00 | $0.00 | **$673.79** | **$0** | **$666.92** | **$240.52** | ***$1,581*** |
| 014 | *PEACH* | $254.37 | $136.56 | $25.04 | $0.00 | $11.38 | **$427.34** | **$0** | **$666.92** | **$2,005.08** | ***$3,099*** |
| 015 | *PEACH* | $118.93 | $136.56 | $25.04 | $0.00 | $34.14 | **$314.67** | **$0** | **$666.92** | **$4,928.00** | ***$5,910*** |
| 017 | *PEACH* | $0.00 | $22.76 | $0.00 | $0.00 | $0.00 | **$22.76** | **$8,677** | **$666.92** | **$0.00** | ***$9,366*** |
| 018 | *PEACH* | $0.00 | $0.00 | $0.00 | $55.00 | $0.00 | **$55.00** | **$0** | **$666.92** | **$540.00** | ***$1,262*** |
| 019 | *PEACH* | $42.70 | $0.00 | $0.00 | $0.00 | $0.00 | **$42.70** | **$9,420** | **$666.92** | **$0.00** | ***$10,130*** |
| 021 | *PEACH* | $283.38 | $45.52 | $25.04 | $132.00 | $0.00 | **$485.94** | **$4,148** | **$666.92** | **$7,558.82** | ***$12,860*** |
| 022 | *PEACH* | $666.43 | $0.00 | $0.00 | $132.00 | $0.00 | **$798.43** | **$0** | **$666.92** | **$1,964.14** | ***$3,429*** |
| 023 | *PEACH* | $128.62 | $182.08 | $0.00 | $0.00 | $0.00 | **$310.70** | **$0** | **$666.92** | **$4,717.85** | ***$5,695*** |
| 025 | *PEACH* | $98.48 | $136.56 | $0.00 | $0.00 | $11.38 | **$246.42** | **$6,899** | **$666.92** | **$461.42** | ***$8,274*** |
| 026 | *PEACH* | $248.21 | $136.56 | $0.00 | $0.00 | $0.00 | **$384.77** | **$0** | **$666.92** | **$917.59** | ***$1,969*** |
| 028 | *PEACH* | $455.21 | $455.21 | $24.23 | $55.00 | $0.00 | **$989.64** | **$0** | **$666.92** | **$3,187.25** | ***$4,844*** |
| 029 | *PEACH* | $159.48 | $159.32 | $0.00 | $0.00 | $22.76 | **$341.57** | **$6,295** | **$666.92** | **$12,220.63** | ***$19,524*** |
| 031 | *PEACH* | $33.69 | $102.42 | $0.00 | $0.00 | $0.00 | **$136.11** | **$0.00** | **$666.92** | **$7,666.86** | ***$8,470*** |
| 002 | *Usual* | $295.88 | $0.00 | $48.45 | $132.00 | $22.76 | **$499.09** | **$9,603** | **$0.00** | **$0.00** | ***$10,102*** |
| 003 | *Usual* | $160.56 | $182.10 | $24.23 | $0.00 | $0.00 | **$366.89** | **$10,605** | **$0.00** | **$0.00** | ***$10,972*** |
| 006 | *Usual* | $159.32 | $0.00 | $0.00 | $0.00 | $0.00 | **$159.32** | **$10,554.17** | **$0.00** | **$0.00** | ***$10,714*** |
| 016 | *Usual* | $383.86 | $307.26 | $75.11 | $0.00 | $0.00 | **$766.23** | **$3,534.96** | **$0.00** | **$0.00** | ***$4,301*** |
| 020 | *Usual* | $534.33 | $0.00 | $0.00 | $0.00 | $0.00 | **$534.33** | **$0.00** | **$0.00** | **$0.00** | ***$534*** |
| 024 | *Usual* | $150.30 | $0.00 | $0.00 | $55.00 | $0.00 | **$205.30** | **$0.00** | **$0.00** | **$0.00** | ***$205*** |
| 027 | *Usual* | $74.07 | $0.00 | $0.00 | $0.00 | $0.00 | **$74.07** | **$6,126.87** | **$0.00** | **$0.00** | ***$6,201*** |
| 032 | *Usual* | $103.70 | $113.80 | $24.23 | $132.00 | $0.00 | **$373.73** | **$0.00** | **$0.00** | **$0.00** | ***$374*** |

**Table S4 Participant-level inpatient stay costs for the PEACH pilot study**

| **ID** | **Treatment arm** | **Total institutional costs for episode of care** | **LOS for episode of care** | **ARDRG code** | **Number of inpatient days during study period** | **Cost per DRG** | **ALOS for DRG** | **Total cost for inpatient stay during the study period** |
| --- | --- | --- | --- | --- | --- | --- | --- | --- |
| 001 | *PEACH* | $0 | - | - | 0 | - | - | $0 |
| 004 | *PEACH* | $0 | - | - | 0 | - | - | $0 |
| 005 | *PEACH* | $0 | - | - | 0 | - | - | $0 |
| 007 | *PEACH* | $6,066 | 1 | J62A | 1 | $4,709 | 5.75 | $819 |
| 008 | *PEACH* | $7,110 | 10 | K62A | 10 | $9,020 | 9.26 | $9,741 |
| 009 | *PEACH* | $0 | - | - | 0 | - | - | $0 |
| 010 | *PEACH* | $1,808 | 3 | G60B | 3 | $2,803 | 3.17 | $2,653 |
| 011 | *PEACH* | $0 | - | - | 0 | - | - | $0 |
| 012 | *PEACH* | $9,440 | 16 | T60A | 11 | $10,495 | 9.12 | $12,658 |
| 013 | *PEACH* | $0 | - | - | 0 | - | - | $0 |
| 014 | *PEACH* | $0 | - | - | 0 | - | - | $0 |
| 015 | *PEACH* | $0 | - | - | 0 | - | - | $0 |
| 017 | *PEACH* | $8,677 | 19 | E62A | 13 | $9,456 | 10.02 | $12,268 |
| 018 | *PEACH* | $0 | - | - | 0 | - | - | $0 |
| 019 | *PEACH* | $9,420 | 2 | E71A | 2 | $9,490 | 8.86 | $2,142 |
| 021 | *PEACH* | $4,148 | 10 | B66B | 8 | $4,292 | 3.88 | $8,849 |
| 022 | *PEACH* | $0 | - | - | 0 | - | - | $0 |
| 023 | *PEACH* | $0 | - | - | 0 | - | - | $0 |
| 025 | *PEACH* | $6,106 | 6 | G60A | 1 | $5,967 | 6.82 | $875 |
| 025 | *PEACH* | $793 | 5 | G60B | 5 | $2,803 | 3.17 | $4,421 |
| 026 | *PEACH* | $0 | - | - | 0 | - | - | $0 |
| 028 | *PEACH* | $0 | - | - | 0 | - | - | $0 |
| 029 | *PEACH* | $6,295 | 8 | I65B | 5 | $4,949 | 4.39 | $5,637 |
| 031 | *PEACH* | $0 | - | - | 0 | - | - | $0 |
| 002 | *Usual* | $9,603 | 32 | E65A | 7 | $6,640 | 7.54 | $6,164 |
| 003 | *Usual* | $10,605 | 43 | B67A | 6 | $12,935 | 13.63 | $5,694 |
| 006 | *Usual* | $10,554 | 51 | J62A | 8 | $4,709 | 5.75 | $6,552 |
| 016 | *Usual* | $3,535 | 3 | B74Z | 3 | $3,157 | 3.06 | $3,095 |
| 020 | *Usual* | $0 | - | - | 0 | - | - | $0 |
| 024 | *Usual* | $0 | - | - | 0 | - | - | $0 |
| 027 | *Usual* | $6,127 | 8 | E71B | 8 | $4,949 | 5.21 | $7,599 |
| 032 | *Usual* | $0 | - | - | 0 | - | - | $0 |

ALOS = average length of stay; ARDRG = Australian Refined Diagnosis Related Group; LOS = length of stay
